# Acute and sub-acute toxicity evaluation of dihydro-*p*-coumaric acid isolated from leaves of *Tithonia diversifolia* Hemsl. A. Gray in BALB/c mice

**DOI:** 10.3389/fphar.2022.1055765

**Published:** 2022-11-23

**Authors:** Thiyam B. Devi, Sarita Jena, Biswajit Patra, Kabrambam D. Singh, Saurabh Chawla, Vishakha Raina, Arunkumar Singh Koijam, Ajay Parida, Yallappa Rajashekar

**Affiliations:** ^1^ Insect Bioresource Laboratory, Animal Bioresources Programme, Institute of Bioresources and Sustainable Development, Department of Biotechnology, Govt. of India, Takyelpat, Manipur, India; ^2^ School of Biotechnology, Kalinga Institute of Industrial Technology, Deemed to be University, Bhubaneswar, Odisha, India; ^3^ Department of Animal House, Institute of Life Sciences, Bhubaneswar, Odisha, India; ^4^ Department of Animal House, School of Biological Sciences, National Institute of Science Education and Research, Bhubaneswar, Odisha, India

**Keywords:** dihydro-p-coumaric acid, Tithonia diversifolia, acute and sub-acute toxicities, BALB/c, pesticides

## Abstract

In present study, the acute and sub-acute toxicities of Dihydro-*p*-coumaric acid isolated from the leaves of *Tithonia diversifolia* (Hemsl.) A. Gray was studied for safety issues in mammals*.* For acute toxicity tests, isolated compound was administered orally in both male and female BALB/c mice at the doses of 200, 800, and 1,600 mg/kg body weight for 7 days. In sub-acute toxicity study 50 and 500 mg/kg bw of the compound was orally administered for 14 days. Toxicity induced behavioural changes, haematological parameters, biochemical markers and histopathological sections were studied after Dihydro-*p*-coumaric acid administration. The vital organs like heart, kidney, uterus and testis revealed no adverse effects at doses of upto 1,600 mg/kg bw and 500 mg/kg bw. Slight hepatotoxicity was however demonstrated by ALT and AST assay but histopathological section did not concur as much. The study demonstrated insignificant difference in the percentage of feed intake, water intake, weight gain, haematological parameters and histopathological changes, with no toxicity signs and mortality. Dihydro-*p*-coumaric acid can be regarded as safe in both acute and sub-acute toxicity assay in both sexes. This indicates Dihydro-*p*-coumaric acid as a viable alternative to synthetic pesticides.

## Introduction

Stored grain insect pests cause serious problems all over the world as they cause reduction of the stored products such as grains, seeds, cereals and legumes quantitatively and qualitatively (Adler et al., 2022). Management of insect pests in storage systems mainly depends upon the use of chemical insecticides like phosphine and methyl bromide. However, the ozone-depleting nature of methyl bromide caused its ban in various countries since 2004. Use of these chemical insecticides continuously leads to certain drawbacks such as developing chemical insecticide resistance to several species, adverse effects on the non-target organisms, formation of pesticidal residue, eradication of economically useful insects, high-cost in production and lastly hazards to the environment including human and wildlife (Rajendran and Sriranjini, 2008; Rajashekar et al., 2013). Plant-based natural pesticides offer a solution to almost all these problems and there is an urgent need to identify and exploit them. In the search for eco-friendly pesticides, research has been performed on plant-based products for protecting crops from insect pests in the field as well as during storage. Bioactive compounds isolated from plants have received special attention for application as botanical pesticides since they show high insecticidal ability, diverse mode of action, non-target organisms and promising safety profiles in mammalian systems. Plants are known for producing bioactive compounds for pesticide synthesis that have been studied as an alternative to plant conventional insecticides (Rattan, 2010; Thormar, 2012). Some plant based compounds such as isoasarone 2, Asaricin 1, and *trans*-asarone 3 isolated from *Piper sarmentosum* roots, pyrethrin from *Chrysanthemum cinerariaefolium,* carvone from *Carum carvi* and azadirachtin from *Azadirachta indica* (Hartmans et al., 1995; Ward et al., 1998; Varma and Dubey 1999; Athanassiou et al., 2005) have gained global attention due to their insecticidal properties against several insect pests. The plant derived insecticides are treated in grains or food commodities and present the risk of residual contamination to consumers, hence requiring a safety assessment (Villas-Boas et al., 2021; Singh et al., 2022). Further, no pesticide is used without any clinical trial before releasing into the market. Azadirachtin (Raizada et al., 2001), linalool, nicotine (Tomizawa et al., 2000), pyrethrin (Verschoyle and Barnes, 1972), and decalside (Rajashekar and Shivanandappa, 2014) were some of the isolated bioactive insecticidal compounds that have been tested for mammalian toxicity.

*Tithonia diversifolia* (Hemsl) A. Gray. known as tree marigold or wild sunflower is a shrub-like perennial or annual plant originating from South and Central America (Carter, 1978; Zhai et al., 2010). It is a known herb used traditionally in agriculture as an insecticide (Castaño-Quintana et al., 2013). *T*. *diversifolia* consist of various bioactive pesticidal compounds, which work together synergistically in controlling insect-pests (Kerebba et al., 2019). We have previously reported that the EO’s of *T. diversifolia* showed insecticidal activity against *T*. *castaneum* and *S*. *oryzae* (Devi et al., 2021). We have isolated a natural biofumigant molecule from *T*. *diversifolia* Hemsl. A Gray leaves identified as dihydro-*p*-coumaric acid that was found to be toxic against stored grain insect pests viz. *R*. *dominica, S*. *oryzae* and *T*. *castaneum* through fumigation activity (Devi et al., 2022). In order to develop dihydro-*p*-coumaric acid derived from *T. diversifolia* leaves as an alternative natural pesticide, investigating its toxicological effect, understanding the dosage, effectiveness level and its adverse effect on man and environment is essential. The current study was undertaken to assess the acute and subacute toxicity of dihydro-*p*-coumaric acid in mammalian system using BALB/c mice.

## Materials and methods

### Dihydro-p-coumaric acid

Dihydro-*p*-coumaric acid was isolated and characterised from methanolic extracts from the leaves of *T. diversifolia* (Devi et al., 2022).

### Animals

In the present study, thirty-one BALB/c mice (6–8 weeks old) of either sex and free of pathogens were used for investigating the toxicity study. All the experimental animals were housed at the Institute of Life Sciences, Bhubaneswar experimental animal facility. Male and female mice were housed separately in an individual matching group in sterile ventilated cages (Citizen Industries, Ahmedabad, India) with wood shredding nesting material, corncob bedding and fed with water *ad libitum* and basic average laboratory rodent diet (VRK Nutritional Solution, India). The animal rearing room was provided with air-conditioning (HVAC) system, a heating, ventilation, and a noise-free environment. Maintenance of BALB/c mice was done at room temperature (22 ± 2°C), a constant relative humidity of 40%–70% under 12 h light and dark cycle. Air changes in the room per hour were 16–20. All experimental animal procedures were strictly conducted with ethical rules, following the Committee for Control and Supervision of Experiments on Animals (CPCSEA) guidelines and approved by the ethical committee from the Institutional Animal Ethics Committee of Institute of Life Sciences, Bhubaneswar (ILS/IAEC-223-AH/APR-21). All the mice selected for the toxicity study were free from bacterial and parasitic pathogens.

### Acute toxicity test

Acute toxicity assay was achieved by using 6–8 weeks old female BALB/c mice according to the OECD (Organization for Economic Co-operation and Development) guideline 423. Sixteen mice were taken in total and were sorted into four groups of four mice each. Dihydro-*p*-coumaric acid was orally administered with a single dose of 1,600, 800, and 200 mg/kg body weight in each group of mice but in the case of the control group, normal drinking water was orally administered to establish the comparative. BALB/c mice were kept devoid of feed for 6 h. Oral administration at the rate of 10 ml/kg body weight of dihydro-*p*-coumaric acid was given to the BALB/c mice by using 18 gauze bent oral gavaging needles. Under same environmental conditions, all the BALB/c mice used in the study were provided with same feed and water. The mice were checked for the first 4 hrs and two times daily (8.00 a.m. and 4.00 p.m.) after administration during the 7 days study period. Various parameters were examined such as the body weight, food consumption, water intake, external appearance including mortality, unusual developments in the skin and eyes, behavioural changes like sleep, coma, lethargy, urination, drowsiness and adverse signs of toxicity like changes in the mucus membrane, salivation, respiratory depression, diarrhoea etc. Body weight changes of the molecule-administered mice were recorded on day-1, day-4, and day-7 whereas left over water volume and feed weight were noted on day-1, day-3, day-5, and day-7 of the experiment.

The mice were weighed on the eighth day of the experiment and sacrificed by using isoflurane inhalation anaesthesia. The blood collection for biochemical assay and haematological study, was done by cardiocentesis for collection of the blood sample. Organs such as liver, heart, spleen and kidney were collected for relative organ weight calculation (Singh et al., 2022).

### Sub-acute toxicity test

Sub-acute toxicity assessment according to the OECD Guideline 407 programme (OECD 407, 2008) was performed using fifteen BALB/c mice. Each sex of mice was randomly separated into three groups of five mice. In the first two groups, both sexes were administered with a dose of 50, 500 mg/kg body weight of dihydro-*p*-coumaric acid while normal drinking water was administered to the control group. Dihydro-*p*-coumaric acid was orally given to the BALB/c mice at a proportion of 10 ml/kg body weight with the use of gauze bent oral gavaging needles. Maintaining under similar feed, water and environmental conditions, all the mice were administered daily with either dihydro*-p*-coumaric acid or normal drinking water for 14 days. Mice were checked and observed carefully for the first 4 h post administration and two times daily (8.00 a.m. and 4.00 p.m.) for a 14 days study period. Mortality, signs of toxicity and toxicity related behavioural changes were among the study parameters. Body weight of the tested BALB/c mice were measured on day-1, day-7, and day-14 whereas weight of the feed and volume of the water consumed were observed on day-1, day-4, day-7, day-11, and day-14 of the study period. The water consumption, feed consumption and change in the percentage of the body weight were also observed and recorded.

At the end of the treatment, all the treated mice were anaesthetized using isoflurane and the blood samples were collected instantly for studying serum biochemical assay and haematological parameters using cardiocentesis. Organs like liver, heart, kidney and uterus from female mice while heart, liver, kidney and testis from male BALB/c mice were collected and weighed for calculating the organ weight and sectioning for histopathological analysis.

### Haematological parameters

Haematological study from blood samples collected in EDTA containing tubes were analyzed using Exigo haematology analyzer. Various parameters like White blood cell (WBC), Red blood cell (RBC), Lymphocyte (LYM), Granulocyte (GRAN), Monocyte (MONO), Haemoglobin (HGB), Haematocrit (HCT), Mean corpuscular haemoglobin (MCH), Mean corpuscular volume (MCV), Mean corpuscular haemoglobin concentration (MCHC), Mean platelet volume (MPV) and Red cell distribution width (RDW) were recorded and analyzed.

### Biochemical assay

Biochemical study was done using a semi-automated biochemistry analyzer (Merilyzer, CliniQuant). For serum collection, centrifugation of the coagulated blood samples at 3,000 rpm at 4^°^C for 10 min was performed. Biochemical parameters viz., alanine aminotransferase (ALT), aspartate aminotransferase (AST) for liver toxicity assessment and creatinine (CRE) for kidney toxicity assessment were measured by using the CliniQuant kits (Singh et al., 2022).

### Histopathological analysis

After euthanasia, histological sections of the liver, heart, kidney, testis and uterus of the BALB/c mice for the treated as well as for control groups were examined. For sub-acute toxicity assay, instead of the spleen, testis (male) and uterus (female) were used. The tissues were fixed in the medium containing 10% neutral buffered formalin and underwent paraffin section of 3–4 µm thickness. The tissues were then stained using haematoxylin and eosin for further histopathological study and were examined using the microscope (Leica microscope, Leica DM500).

### Relative organ weight analysis

The calculation for Relative organ weight (ROW) of each animal was performed using the formula, relative Organ weight (%) = (organ weight/body weight) × 100 (Hor et al., 2012).

### Statistical analysis

All the data were expressed as mean ± standard error (SEM). One-way ANOVA analysis was performed using GraphPad prism 8.4.0 and Tukey test post-hac was used to determine statistical significance among groups. A *p*-value of ≤0.05 was considered statistically significant.

## Results

### Acute toxicity

The acute toxicity study of Dihydro-*p*-coumaric acid isolated from *T. diversifolia* revealed that all the BALB/c mice treated at different doses were healthy, active, and showed no signs of toxicity. The isolated bioactive compound at the highest dose of 1,600 mg/kg body weight did not show any behavioural changes in all the treated mice. Normal external appearance with no changes in eyes and skin was observed in all the dihydro-*p*-coumaric acid treated mice. Sleep and urination were normal and there was no sign of lethargy, coma, and drowsiness among the treated mice. The body weight of the treated mice (200, 800, and 1,600 mg/kg bw) displayed no significant changes relating to dihydro-*p*-coumaric acid treatment when compared with the control mice (*p* > 0.05). Significant changes in food and water intake in dihydro-*p*-coumaric acid treated mice was not observed (*p* > 0.05) (Table 1). The relative organ weight of heart, kidney and spleen were indifferent to that of control group. However, the relative organ weight of liver was found to be significantly different at the doses of 200 mg/kg and 1,600 mg/kg but not 800 mg/kg body weight.

**TABLE 1:** Effect of the Dihydro-*p*-coumaric acid on body weight, food intake and water intake on BALB/c mice in acute toxicity study.

Treatment	Initial weight	Day 4 (g)	Day 7 (g)	Food intake (g/day/mice)	Water intake (ml/day/mice)
Control	15.42 ± 0.36^a^	16.70 ± 0.24^a^	16.62 ± 0.27^a^	5.45 ± 0.20^a^	3.41 ± 0.09^c^
200 mg/kg bw	16.10 ± 0.73^a^	17.02 ± 0.91^a^	16 92 ± 0.63^a^	5.85 ± 0.19^b^	2.20 ± 0.10^a^
800 mg/kg bw	17.65 ± 1.27^a^	18.12 ± 1.30^b^	18.50 ± 1.25^b^	5.88 ± 0.12^b^	3.0 ± 0.20^b^
1,600 mg/kg bw	17.0 ± 0.81^a^	17.82 ± 0.71^b^	18.0 ± 0.68^b^	6.09 ± 0.30^b^	2.40 ± 0.10^a^

Values are expressed as mean ± SEM (df = 3) followed by the same letter of the alphabet (a, b, c) do not differ significantly each other (ANOVA, Tukey test post-hoc, *p* > 0.05).

### Sub-acute toxicity

No adverse treatment-related change in behaviour of both sexes of BALB/c mice treated with dihydro-*p*-coumaric acid was observed when compared to the controls. Mortality was not observed in the compound-treated groups as well as in control mice. The consumption of feed was negligibly lower in the treated female BALB/c mice (2.50 and 2.51 g/day/mice for 50 and 500 mg/kg bw respectively) as against the controls (2.54 g/day/mice) (Table 2). There was no significant difference in all the treated groups of both sexes (*p* > 0.05). Change in water intake of both the sexes of treated mice was insignificant except for 500 mg/kg bw treated males (*p* < 0.05) as presented in Table 2. Only 500 mg/kg bw treated males also showed negligible significance in average percentage body weight gain among while the rest treatment groups in both sexes was insignificant (Table 2). Among females, the percentage weight gains were 11.26%, 10.24%, and 7.97% respectively for control, 50 mg/kg bw and 500 mg/kg bw treated BALB/c mice.

**TABLE 2:** Effect of the Dihydro-*p*-coumaric acid on body weight, food intake and water intake on BALB/c mice in sub-acute toxicity study.

	Female					Male				
	Initial weight	Day 7 (g)	Day 14 (g)	Food intake (g/day/mice)	Water intake (ml/day/mice)	Initial weight	Day 7 (g)	Day 14 (g)	Food intake (g/day/mice)	Water intake (ml/day/mice)
Control	19.18 ± 0.31^b^	18.62 ± 0.87^a^	21.34 ± 0.35^b^	2.54 ± 0.13^a^	3.69 ± 0.09^c^	16.10 ± 0.88^a^	16.82 ± 0.92^a^	17.94 ± 0.70^a^	2.82 ± 0.49^a^	4.03 ± 0.06^c^
50 mg/kg bw	18.36 ± 0.74^a^	19.48 ± 0.69^b^	20.24 ± 0.64^a^	2.50 ± 0.08^a^	2.81 ± 0.02^a^	17.08 ± 0.47^b^	17.62 ± 0.42^a^	18.48 ± 0.40^a^	3.68 ± 0.40^b^	3.69 ± 0.09^b^
500 mg/kg bw	19.32 ± 0.52^a^	20.04 ± 0.28^b^	20.86 ± 0.33^a^	2.51 ± 0.06^a^	2.94 ± 0.16^a^	16.54 ± 0.68^a^	18.76 ± 0.05^b^	17.72 ± 0.73^a^	2.98 ± 0.24^a^	3.42 ± 0.13^a^

Values are expressed as mean ± SEM (df = 4) followed by the same letter of the alphabet (a, b) do not differ significantly each other (ANOVA, Tukey test post-hoc, *p* > 0.05).

### Haematological parameters

In the acute toxicity test, all blood parameters viz. WBC, LYM, GRAN, MONO, HGB, HCT, RBC, MCV, MPV, and RDW, except MCH and MCHC of the treated mice did not demonstrated any significant changes on comparison to the controls (Table 3). MCH showed moderate significance in 200 mg/kg bw and 800 mg/kg bw but not 1,600 mg/kg bw treated mice. MCHC showed negligible significance (*p* = 0.0473) in all the treated groups. In the sub-acute toxicity test, males and females displayed differential responses. In males, all blood parameters except MCV (*p* < 0.05) in both treatment groups showed no significant difference compared to controls. Females on the other hand displayed insignificant difference in both treatment groups compared to controls, for blood parameters viz. WBC, LYM, MONO, GRAN, MCH, MCHC, and MPV (Table 4). HGB, HCT, and RBC showed mildly significant increase in the 500 mg/kg bw treated mice but not in 50 mg/kg bw treated mice. RDW showed significant increase in both treatment groups in females. MCV displayed mild significance in 50 mg/kg bw treated mice but not 500 mg/kg bw treated mice.

**TABLE 3:** Effect of the oral administration of dihydro *p*-coumaric acid haematological parameters in acute toxicity studies.

Parameters	Unit	Normal group	200 mg/kg BW	800 mg/kg BW	1,600 mg/kg BW
White bool cell (WBC)	10^9^/l	6.55 ± 0.19^c^	5.77 ± 0.32^b^	6.65 ± 0.30^c^	3.92 ± 0.53^a^
Lymphocyte (LYM)	%	81.47 ± 1.15^b^	80.12 ± 1.6^b^	80.1 ± 0.46^b^	78.27 ± 1.20^a^
Monocyte (MONO)	%	5.6 ± 0.56^a^	6.1 ± 0.41^b^	5.17 ± 0.20^a^	6.52 ± 0.49^c^
Granulocyte (GRAN)	10^9^/l	0.83 ± 0.1^c^	0.75 ± 0.04^b^	0.95 ± 0.06^d^	0.55 ± 0.06^a^
Granulocyte (GRAN)	%	12.9 ± 1.15^a^	13.7 ± 1.18^a^	14.72 ± 0.41^b^	15.2 ± 0.71^b^
Haemoglobin (HGB)	g/dl	17.3 ± 0.19^a^	16.7 ± 0.81^a^	17.55 ± 0.18^0^	17.42 ± 0.23^a^
Haematocrit (HCT)	%	44.85 ± 0.03^a^	44.75 ± 0.12^a^	46.85 ± 0.29^a^	46.55 ± 0.25^a^
Red blood cell (RBC)	10^12^/l	9.26 ± 0.06^b^	9.20 ± 0.40^b^	7.34 ± 1.45^a^	9.43 ± 0.09^b^
Mean corpuscular volume (MCV)	fl	48.38 ± 0.35^b^	48.55 ± 0.22^b^	47.5 ± 0.59^a^	49.37 ± 0.42^c^
Mean corpuscular haemoglobin (MCH)	Pg	18.68 ± 0.13^c^	18.12 ± 0.10^b^	17.7 ± 0.08^a^	18.5 ± 0.16^c^
Mean corpuscular haemoglobin concentration (MCHC)	g/dl	38.63 ± 0.39^b^	37.35 ± 0.10^a^	37.47 ± 0.33^a^	37.42 ± 0.34^a^
Red cell distribution width (RDW)	%	17.73 ± 0.32^b^	17.07 ± 0.10^a^	17.02 ± 0.33^a^	17.37 ± 0.30^b^
Mean platelet volume (MPV)	fl	4.73 ± 0.22^a^	4.8 ± 0.19^a^	5.25 ± 0.14^b^	4.9 ± 0.04^a^

Values are expressed as mean ± SEM (df = 3) followed by the same letter of the alphabet (a, b, c) do not differ significantly each other (ANOVA, Tukey test post-hoc, *p* > 0.05).

**TABLE 4:** Effect of the oral administration of dihydro-*p*-coumaric acid on haematological parameters in the sub-acute toxicity studies.

Parameters	Unit	Normal group	50 mg/kg bw enriched fraction	500 mg/kg bw enriched fraction
Male				
White bool cell (WBC)	10^9^/l	6.86 ± 1.01^b^	4.97 ± 0.42^a^	7.04 ± 0.43^b^
Lymphocyte (LYM)	%	79.36 ± 0.90^a^	81.1 ± 1.57^b^	81.28 ± 1.59^b^
Monocyte (MONO)	%	4.12 ± 0.34^a^	4.52 ± 0.27^a^	4.28 ± 0.26^a^
Granulocyte (GRAN)	10^9^/l	1.06 ± 0.13^b^	0.7 ± 0.10^a^	1.02 ± 0.08^b^
Granulocyte (GRAN)	%	16.52 ± 1.14^b^	14.37 ± 1.37^a^	14.7 ± 1.39^a^
Haemoglobin (HGB)	g/dl	16.24 ± 1.56^a^	18.17 ± 1.20^b^	19.86 ± 0.79^c^
Haematocrit (HCT)	%	44.18 ± 3.87^a^	49.67 ± 3.72^b^	52.16 ± 1.67^c^
Red blood cell (RBC)	10^12^/l	8.94 ± 0.84^a^	10.37 ± 0.77^b^	10.73 ± 0.32^b^
Mean corpuscular volume (MCV)	Fl	49.5 ± 0.43^b^	47.85 ± 0.35^a^	47.9 ± 0.12^a^
Mean corpuscular haemoglobin (MCH)	Pg	18.12 ± 0.15^b^	17.67 ± 0.68^a^	18.28 ± 0.16^b^
Mean corpuscular haemoglobin concentration (MCHC)	g/dl	36.66 ± 0.48^a^	36.9 ± 1.34^a^	38.28 ± 0.34^b^
Red cell distribution width (RDW)	%	18.26 ± 0.93^b^	17.55 ± 0.24^a^	18.44 ± 0.28^b^
Mean platelet volume (MPV)	Fl	4.54 ± 0.18^a^	5.2 ± 0.22^b^	4.64 ± 0.17^a^
**Female**				
White bool cell (WBC)	10^9^/l	5.8 ± 0.59^b^	4.52 ± 0.43^a^	5.76 ± 0.30^b^
Lymphocyte (LYM)	%	80.72 ± 1.67^b^	77.1 ± 1.30^a^	78.8 ± 0.86^a^
Monocyte (MONO)	%	5.18 ± 0.12^c^	4.94 ± 0.22^b^	4.66 ± 0.12^a^
Granulocyte (GRAN)	10^9^/l	0.78 ± 0.04^a^	0.78 ± 0.10^a^	0.9 ± 0.05^b^
Granulocyte (GRAN)	%	14.1 ± 1.30^a^	17.96 ± 1.15^c^	16.48 ± 0.95^b^
Haemoglobin (HGB)	g/dl	17.52 ± 0.70^a^	18.24 ± 0.64^b^	20.32 ± 0.55^c^
Haematocrit (HCT)	%	44.84 ± 2.10^a^	46.36 ± 1.94^b^	52.56 ± 1.43^c^
Red blood cell (RBC)	10^12^/l	9.24 ± 0.41^a^	9.7 ± 0.36^a^	10.79 ± 0.27^b^
Mean corpuscular volume (MCV)	fl	48.44 ± 0.12^b^	47.76 ± 0.24^a^	48.66 ± 0.11^b^
Mean corpuscular haemoglobin (MCH)	Pg	18.9 ± 0.18^a^	18.82 ± 0.04^a^	18.8 ± 0.1^a^
Mean corpuscular haemoglobin concentration (MCHC)	g/dl	39.16 ± 0.4^b^	39.42 ± 0.29^b^	38.62 ± 0.15^a^
Red cell distribution width (RDW)	%	17.78 ± 0.25^a^	20.12 ± 0.29^c^	19.3 ± 0.17^b^
Mean platelet volume (MPV)	fl	5.24 ± 0.08^b^	5.04 ± 0.09^a^	5.1 ± 0.05^a^

Values are expressed as mean ± SEM (df = 4) followed by the same letter of the alphabet (a, b, c) do not differ significantly each other (ANOVA, Tukey test post-hoc, *p* > 0.05).

### Serum biochemistry analysis

**FIGURE 1:**
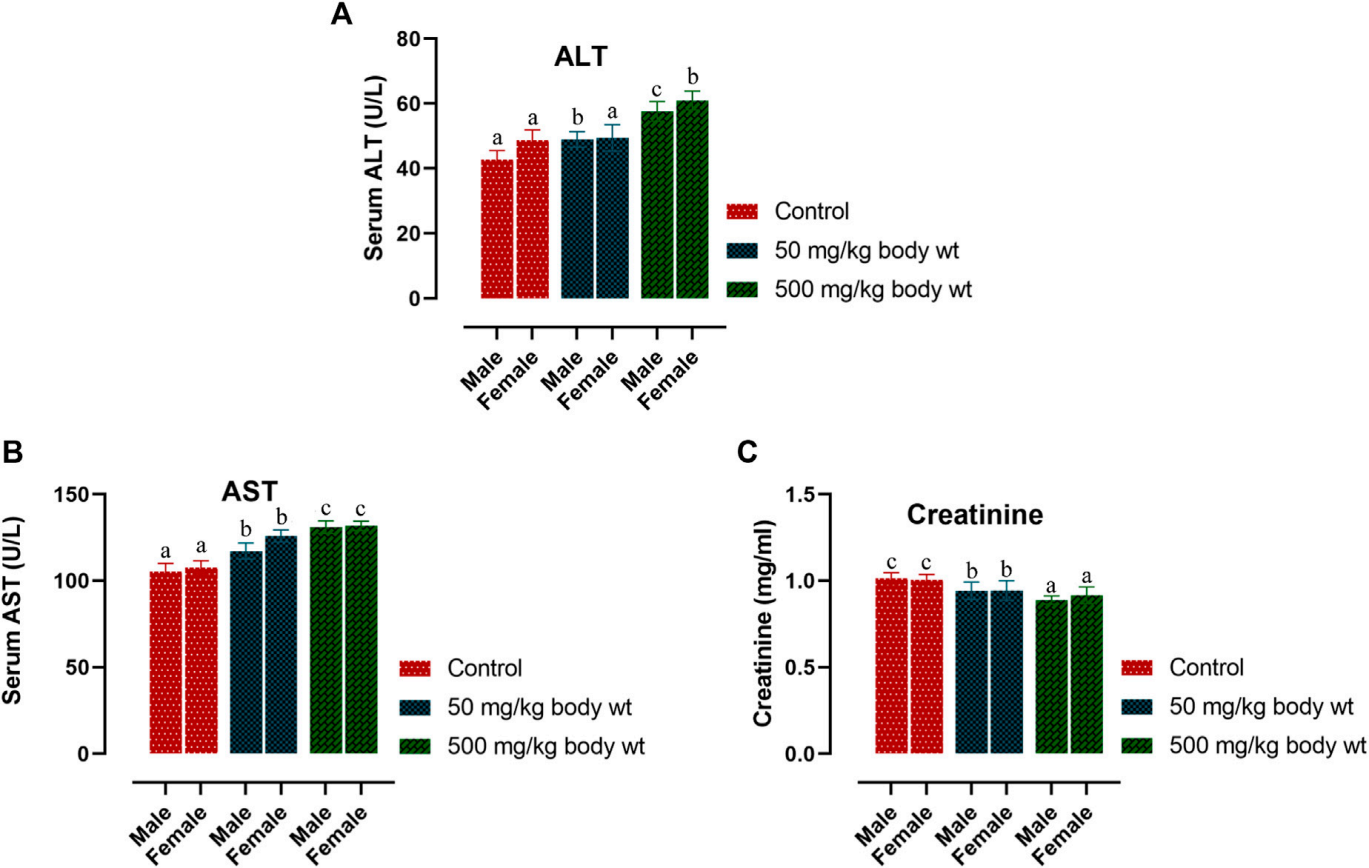
Effect of dihydro-*p*-coumaric acid on biochemical parameters depicting liver and kidney function on BALB/c mice viz., alanine aminotransferase (ALT), aspartate aminotransferase (AST), and creatinine (CRE) Significance level set at 0.05. Values are expressed as mean ± SEM followed by the same letter of the alphabet **(A–C)** do not differ significantly each other (ANOVA, Tukey test post-hoc, *p* > 0.05).

Serum biochemistry analysis of three parameters relating to hepatocellular activity and renal function viz. Aspartate aminotransferase (AST), Alanine aminotransferase (ALT) and Creatinine (CRE) are reported in Figure 1. In both the doses, there was a slight increase in the ALT level of treated males (*p < 0.05*). Treated female mice showed significant changes in ALT level only at the higher dose (*p* < 0.05) but not at the lower dose. Serum AST levels in both sexes were observed to increase significantly in all treated groups as against the controls. (males 50 mg/kg bw-*p* < 0.005; all other groups-*p* < 0.001). Serum CRE levels lowered in treated groups but a significant difference was observed only with the higher dose treated individuals of both sexes. (males 500 mg/kg bw-*p* < 0.005; females 500 mg/kg bw-*p* < 0.05).

### Histopathological analysis

**FIGURE 2:**
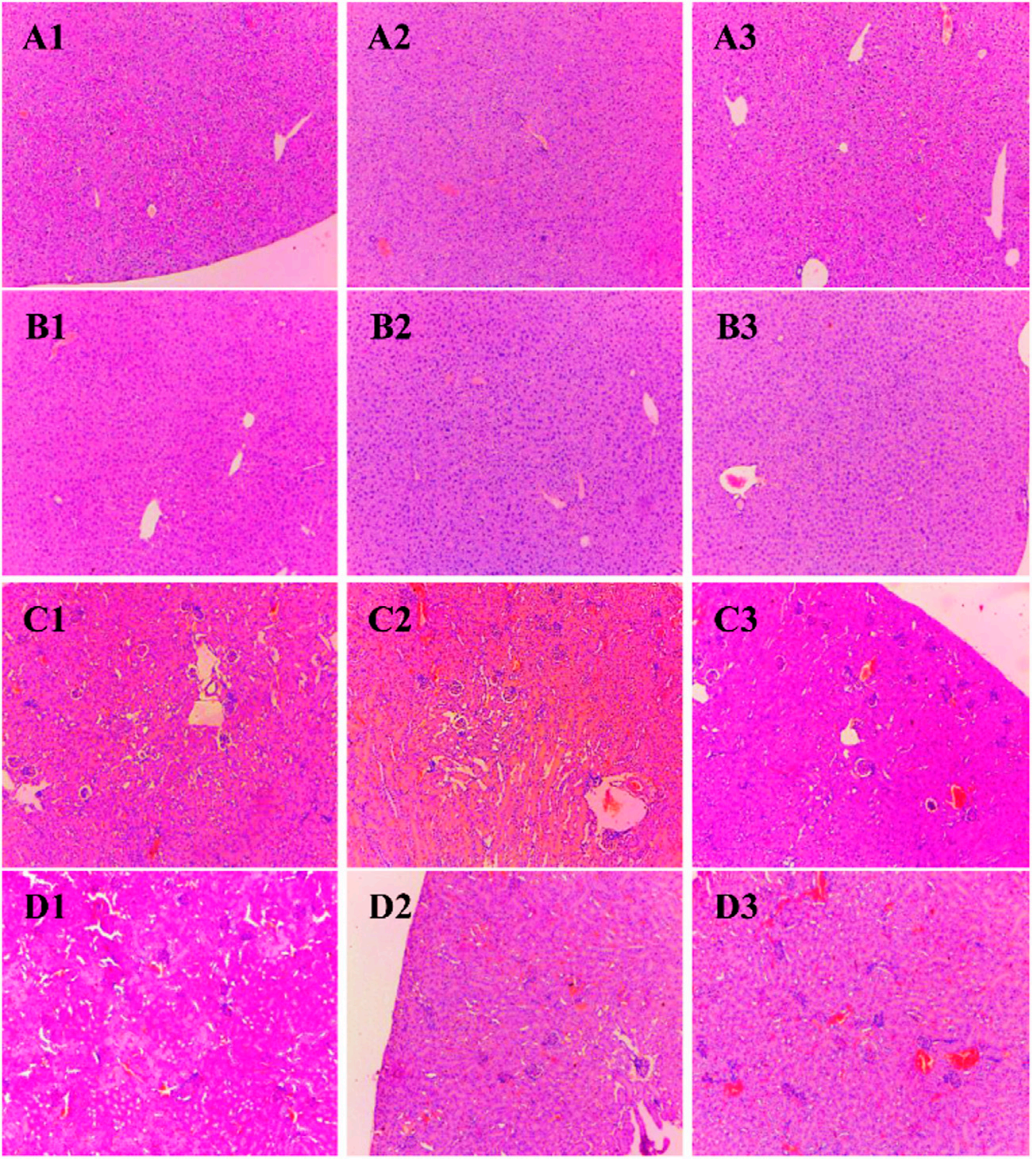
Photomicrograph of vital organs section in BALB/c mice viz., Liver and kidney of BALB/c mice administered with distilled water and dihydro-*p*-coumaric acid for 14 days. **(A1)**: Liver of female mice treated with distilled water only, **(A2)**: Liver of female mice treated with 50 mg/kg body weight dihydro-*p*-coumaric acid, **(A3)**: Liver of female mice treated 500 mg/kg body weight dihydro-*p*-coumaric acid. **(B1)**: Liver of male mice treated with distilled water only, **(B2)**: Liver of male mice treated with 50 mg/kg body weight dihydro-*p*-coumaric acid, **(B3)**: Liver of male mice treated with 500 mg/kg body weight dihydro-*p*-coumaric acid. **(C1)**: Kidney of female mice treated with distilled water only, **(C2)**: Kidney of female mice treated with 50 mg/kg body weight dihydro-*p*-coumaric acid, **(C3)**: Kidney of female mice treated with 500 mg/kg body weight dihydro-*p*-coumaric acid. **(D1)**: Kidney of male mice treated with distilled water only, **(D2)**: Kidney of male mice treated with 50 mg/kg body weight dihydro-*p*-coumaric acid, **(D3)**: Kidney of male mice treated with 500 mg/kg body weight dihydro-*p*-coumaric acid.

**FIGURE 3:**
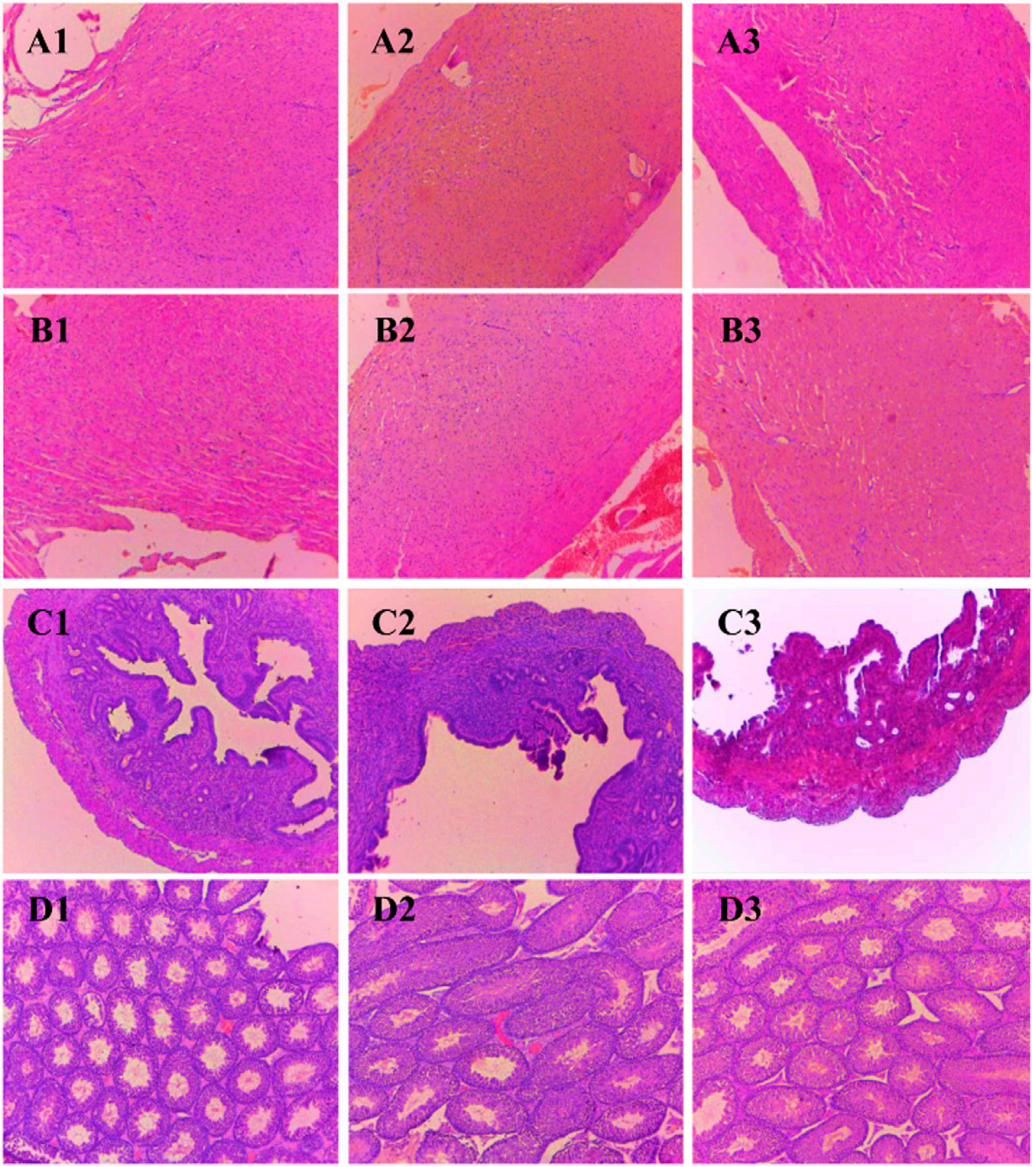
Photomicrograph of vital organs section in BALB/c mice viz., Heart, Uterus and Testis of BALB/c mice administered with distilled water and dihydro-*p*-coumaric acid for 14 days. A1: Heart of female mice treated with distilled water only, A2: Heart of female mice treated with 50 mg/kg body weight dihydro-*p*-coumaric acid, A3: Heart of female mice treated 500 mg/kg body weight dihydro-*p*-coumaric acid. B1: Heart of male mice treated with distilled water only, B2: Heart of male mice treated with 50 mg/kg body weight dihydro-*p*-coumaric acid, B3: Heart of male mice treated with 500 mg/kg body weight dihydro-*p*-coumaric acid. C1: Uterus of female mice treated with distilled water only, C2: Uterus of female mice treated with 50 mg/kg body weight dihydro-*p*-coumaric acid, C3: Uterus of female mice treated with 500 mg/kg body weight dihydro-*p*-coumaric acid. D1: Testis of male mice treated with distilled water only, D2: Testis of male mice treated with 50 mg/kg body weight dihydro-*p*-coumaric acid, D3: Testis of male mice treated with 500 mg/kg body weight dihydro-*p*-coumaric acid.

Histological sections of the liver, heart, kidney, uterus, and testis of all BALB/c mice involved in the study were analyzed (Figures 2, 3). Microscopic observation depicted slight histopathological change in the liver (male) and kidney (male and female) in the tested BALB/c mice after administrating dihydro-*p*-coumaric acid whereas no histopathological variations were observed in heart, uterus. The comprehensive reports of five vital organ tissue sections are as follows: the histo-architecture of female liver administered with the lowest dose and highest dose was indistinguishable from that of the control (Figures 2A1–A3). No apoptosis or necrosis was observed. The liver in 50 mg/kg bw treated male mice were indifferent from the controls. However, 500 mg/kg bw treated mice displayed modest lesion. In case of kidney, no distinct differences have been observed between the sections of control and treated mice of both sexes. (Figures 2C1–C3 and Figures 3D1–D3). Bowman’s capsule remains relatively unaffected. Glomerular organisation, distal tubules, collecting ducts and interspersed smooth muscles are relatively normal and comparable to control. Apoptosis or necrotic lesions are indistinct. However, hyperemia has been observed more in the mice treated with 500 mg/kg bw and even more prominently in males compared to controls. The heart sections displayed no histopathological changes (Figures 3A1–A3 and Figures 3B1–B3). Apoptotic or necrotic lesions were not observed in both sexes of all the treated mice. The uterus in female mice was unaffected in the treated groups and similar to controls. All layers of uterus particularly endometrial layer displayed no unusual patterns and remain healthy. (Figures 3C1–C3). The testis section in treated males was similar to the controls. Seminiferous tubules, Sertoli cells and leydig’s cells were observed to be intact with no necrotic lesions or apoptosis (Figures 3D1–D3).

### Relative organ weight analysis

**FIGURE 4:**
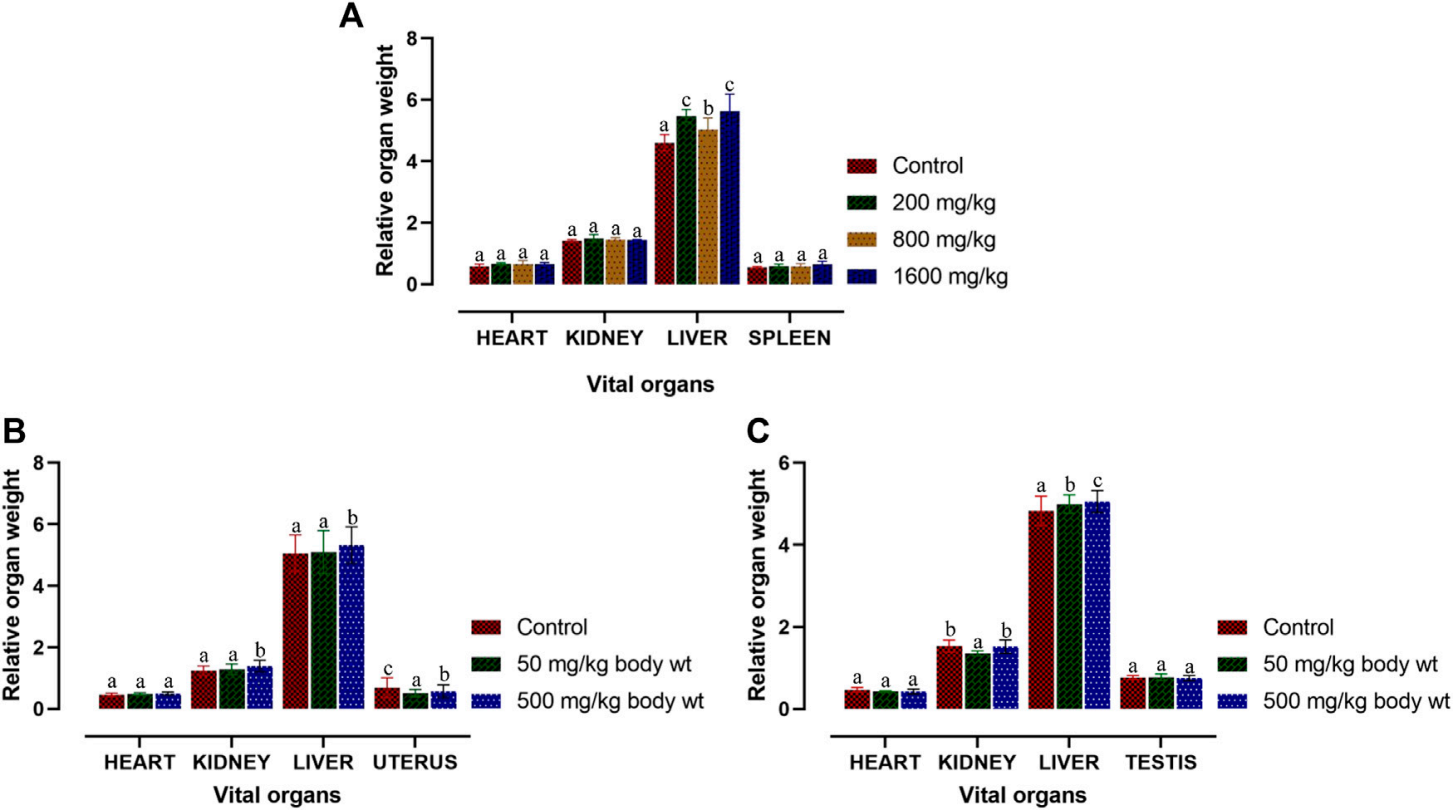
Relative organ weight of BALB/c mice post acute and sub-acute treatment of dihydro-*p-*coumaric acid. Values are expressed as mean ± SEM followed by the same letter of the alphabet **(A–C)** do not differ significantly each other (ANOVA, Tukey test post-hoc, *p* > 0.05).

Macroscopic analysis was performed for the vital organs in both treated and control BALB/c mice. Relative organ weight (ROW) in acute and sub-acute toxicity test is displayed in Figures 4A–C. In acute-toxicity study, there was a significant difference in the relative organ weight of liver at the doses of 200 mg/kg and 1,600 mg/kg bw but not 800 mg/kg bw. Changes in the relative organ weight of all the other compound treated vital organs of BALB/c mice were insignificant in acute-toxicity study (Figure 4A). Similarly, in sub-acute toxicity study, the relative organ weight of heart, kidney, liver, uterus and testis treated with 50 mg/kg and 500 mg/kg bw did not showed any significant change (Figures 4B,C).

## Discussions

Despite its invasive nature, *T. diversifolia* is utilised in many traditional folklores as an anti-repellent and an insecticide. Keeping in view of the various disadvantages of synthetic pesticides, an attempt has been made to generate an eco-friendly insecticide that is of plant origin and can act as a stored grain protectant. Evaluating its safety issue in the mammalian system for potential release in the global market is thus initiated for dihydro-*p*-coumaric acid derived from *T. diversifolia*. Our earlier report has also revealed that dihydro-*p*-coumaric acid isolated from the methanolic extract of *T. diversifolia* leaves (Devi et al., 2022) has shown biofumigant activity against *Sitophilus oryzae* L, *Rhyzopertha dominica* F *and Tribolium castaneum Herbst*. Dihydro-*p*-coumaric acid has limited reports on mammalian toxicity study and thus needed further validation by scientific investigations. In a study (Adebayo et al., 2009), the aqueous extracts of *T. diversifolia* leaves on the Wistar rats when orally administered showed toxicity effect at the dosage of 100 mg/kg and 200 mg/kg. In another study (Elufioye et al., 2009), 70% ethanolic extract derived from the aerial parts of *T*. *diversifolia* when administered orally to Wistar rats, showed nephron and hepato-toxicity when it was tested with the minimum dose. There are very few reports documented on the toxicological effects of this plant (Elufioye and Agbedahunsi, 2004). The present experiment aims in assessing the acute and sub-acute toxicity of dihydro-*p*-coumaric acid on BALB/c mice by oral administration. Acute and sub-acute toxicity tests did not showed any visible salivation or symptoms of respiratory depression and diarrhoea. In the acute toxicity test, there was a significant reduction in water intake in 200 mg/kg bw and 1,600 mg/kg bw treated mice but not in 800 mg/kg bw treated mice. Only males treated with 500 mg/kg bw of compound in the sub acute toxicity test showed negligibly significant decrease in average percentage weight gain and water intake. This may be attributed to the decreased average percentage weight gain as well as other confounding factors.

Further, changes in the blood parameters of the compound treated BALB/c mice displayed differential significance in both acute and sub-acute toxicity tests. A common manifestation in the compound treated mice was increased in RBC related physiology that likely points to anaemic condition in female but not in male. The increase in haematocrit percentage is likely due to dehydration as demonstrated by their decrease water intake. Inspite of such changes, there was least effects on external appearance, food consumption, behavioural changes or signs of toxicity. Water intake and feed consumption was slightly lower in the case of treated female BALB/c mice in sub-acute toxicity study. Assessment of ROW of heart, kidney, uterus and testis after both acute and sub-acute treatment displayed no significant difference between all treated mice and controls. The difference between ROW of liver after acute and sub-acute treatment, may be attributed to the high dose in acute treatment and a likely fast regenerative potential of liver post toxic exposure. Traesel et al. (2014) marked the importance of enzymes such as ALT, AST as sensitive biomarkers of hepatocellular activity. The level of the enzymes, ALT and AST increases when there is damage in the liver *via* leading to liver necrosis, hepatitis, and liver toxicity. The three parameters of serum biochemistry analysis viz Alanine aminotransferase (ALT), Aspartate aminotransferase (AST) and Creatinine (CRE) displayed significant difference between treated mice and controls of both sexes at the higher dose of 500 mg/kg bw. At the lower dose of 50 mg/kg bw, ALT did not increased significantly in females and this may be attributed to female specific hormonal physiology particularly relating to estrus cycle. Serum AST levels increased significantly in all treated groups. The ALT and AST analysis reveals certain level of toxicity in mice and is in conjunction with an increased ROW of liver among acutely treated mice (Stojanović et al., 2019; Owojuyigbe et al., 2022). The modest lesions observed in 500 mg/kg bw treated mice also indicate slight toxicity of Dihydro-*p*-coumaric acid treatment. In spite of these mild to moderate effects of Dihydro-*p*-coumaric acid on liver, the observation of no significant change in the behavioural parameters particularly feeding or water intake in treated mice indicate that there is no adverse effects due to treatment. Serum Creatinine levels lowered with Dihydro-*p*-coumaric acid treatment but were not significant indicating little or no nephrotoxicity. The same is also supported by the histological section of kidney. Further, the presence of dihydro-*p*-coumaric acid has been reported in olive (Boskou, 2010) which has long been consumed by man, indicating that there is little or no adverse effects on consumption by man. Based on the results of the current investigation, dihydro-*p*-coumaric acid shows little or no effect at both low and high doses to vital organs other than liver in BALB/c mice of both the sexes. There is further need to study the retention time and degradation process of dihydro-*p*-coumaric acid in nature. This will allow the effective utilization of dihydro-*p*-coumaric acid as the next viable alternative to synthetic pesticides.

## Conclusion

The findings of the present study involving acute and sub-acute toxicity tests of dihydro-*p*-coumaric acid using BALB/c mice demonstrated no adverse effects at behavioural, biochemical and histopathological levels or mortality even at the highest dose of 1,600 mg/kg bw and 500 mg/kg bw. Biochemical analysis revealed mild changes in hepatic activity biomarkers ALT and AST while histopathological investigation on liver denoted mild hepatotoxic effect. Histopathological investigation on other vital organs such as heart, kidney, uterus and testis revealed no adverse or observable effects at the lower as well as higher doses of upto 1,600 mg/kg bw and 500 mg/kg bw. This clearly indicates the potential of dihydro-*p*-coumaric acid as a viable alternative to synthetic pesticides.

## Data Availability

The original contributions presented in the study are included in the article/supplementary materials, further inquiries can be directed to the corresponding author.

## Abbreviations

ALT, Alanine aminotransferase; AST, Aspartate aminotransferase; CRE, Creatinine; GRAN, Granulocyte; HCT, Haematocrit; HGB, Haemoglobin; LYM, Lymphocyte; MCH, Mean corpuscular haemoglobin; MCHC, Mean corpuscular haemoglobin concentration; MCV, Mean corpuscular volume; MONO, Monocyte; MPV, Mean platelet volume; RBC, Red blood cell; RDW, Red cell distribution width; WBC, White bool cell.
