# Supplementary figures and images for: Acute and sub-acute toxicity evaluation of dihydro-p-coumaric acid isolated from leaves of Tithonia diversifolia Hemsl. A. Gray in BALB/c mice
Source: Front Pharmacol. 2022 Nov 23;13:1055765. doi: 10.3389/fphar.2022.1055765 (PMC9727178; doi:10.3389/fphar.2022.1055765)

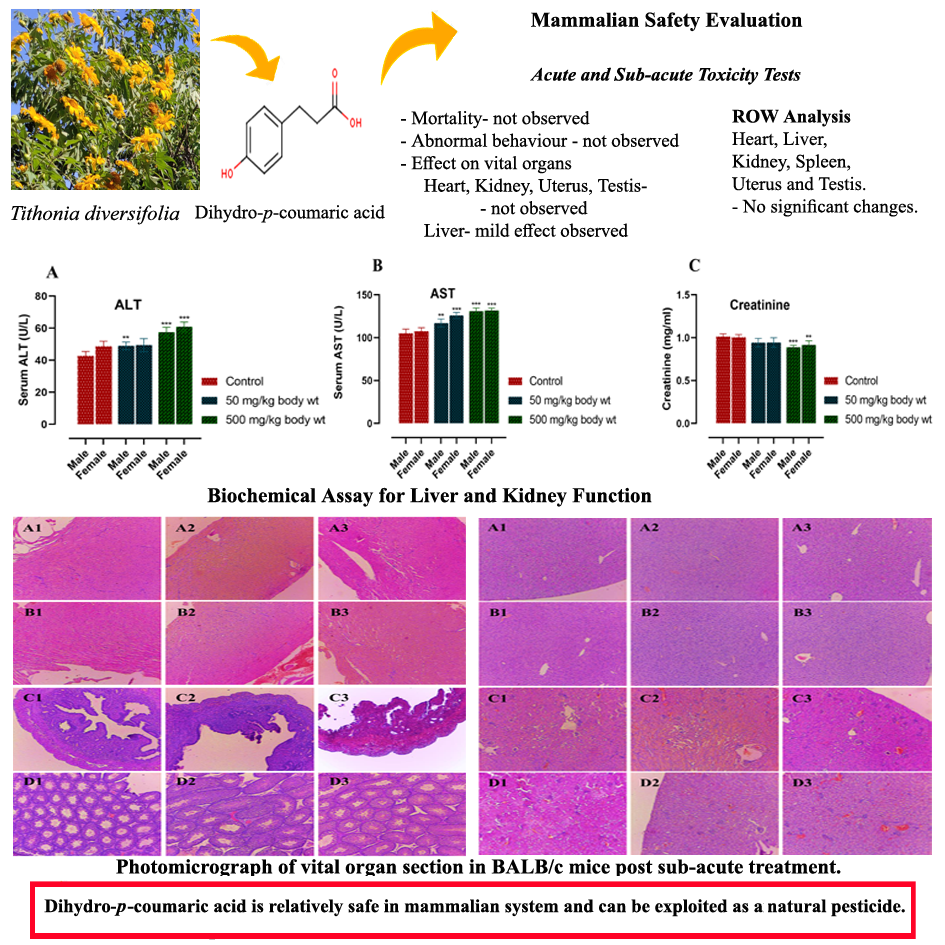

Supplement: Supplementary file 1 [file Image1.TIF]
